# Pulmonary Tumor Thrombotic Microangiopathy Caused by Esophageal Adenocarcinoma: A Case Report

**DOI:** 10.7759/cureus.44435

**Published:** 2023-08-31

**Authors:** James O Peeples, Joshua King, Victoria Chung, Lane Maley, Jonathan Mizrahi

**Affiliations:** 1 Internal Medicine, Ochsner Clinic Foundation, New Orleans, USA; 2 Hematology/Oncology, Ochsner Clinic Foundation, New Orleans, USA; 3 Gastrointestinal Oncology, Ochsner Clinic Foundation, New Orleans, USA

**Keywords:** esophageal cancer (ec), adenocarcinoma, pulmonary hypertension, right-sided heart failure, pulmonary tumor thrombotic microangiopathy

## Abstract

Pulmonary tumor thrombotic microangiopathy (PTTM ) is a rare condition of uncertain incidence given its likely underdiagnosis. PTTM has been described most frequently in association with gastric adenocarcinoma, but other primary malignancies have been identified. The prognosis of PTTM is very poor, and patients often die within days or weeks of diagnosis. There are, however, several medications currently being used with unknown therapeutic benefits. The case presented below describes a patient with PTTM and esophageal adenocarcinoma, which may be the first report of its kind. One other case of esophageal cancer associated with PTTM was found in the literature review, but it is of squamous cell carcinoma histology. Herein, we report a case of a male with rapidly progressive pulmonary hypertension and right heart failure who, in the course of treatment/evaluation, was found to have esophageal adenocarcinoma. While early diagnosis may not alter the course of the disease, antemortem diagnosis may identify better therapeutic options and better inform patients of their prognosis, allowing them to maintain autonomy in their medical decisions.

## Introduction

Pulmonary tumor thrombotic microangiopathy (PTTM) is a rare, under-reported, and poorly studied condition in which tumor cells embolize to the pulmonary microvasculature, resulting in activation of the coagulation cascade and fibrocellular intimal proliferation [1]. These fibrotic changes lead to severe pulmonary hypertension, rapidly progressive right heart failure, and, eventually, death. In a study that performed autopsies on patients with known carcinoma, the incidence of PTTM was found to be 1.4% [2]. The challenge remains the low rate of ante-mortem diagnosis, as nearly 80% of identified cases are diagnosed by autopsy [3]. The purpose of this presentation is to increase awareness and earlier identification of this condition in patients with rapidly progressive right heart failure, pulmonary hypertension, and malignancy.

## Case presentation

**Figure 1:**
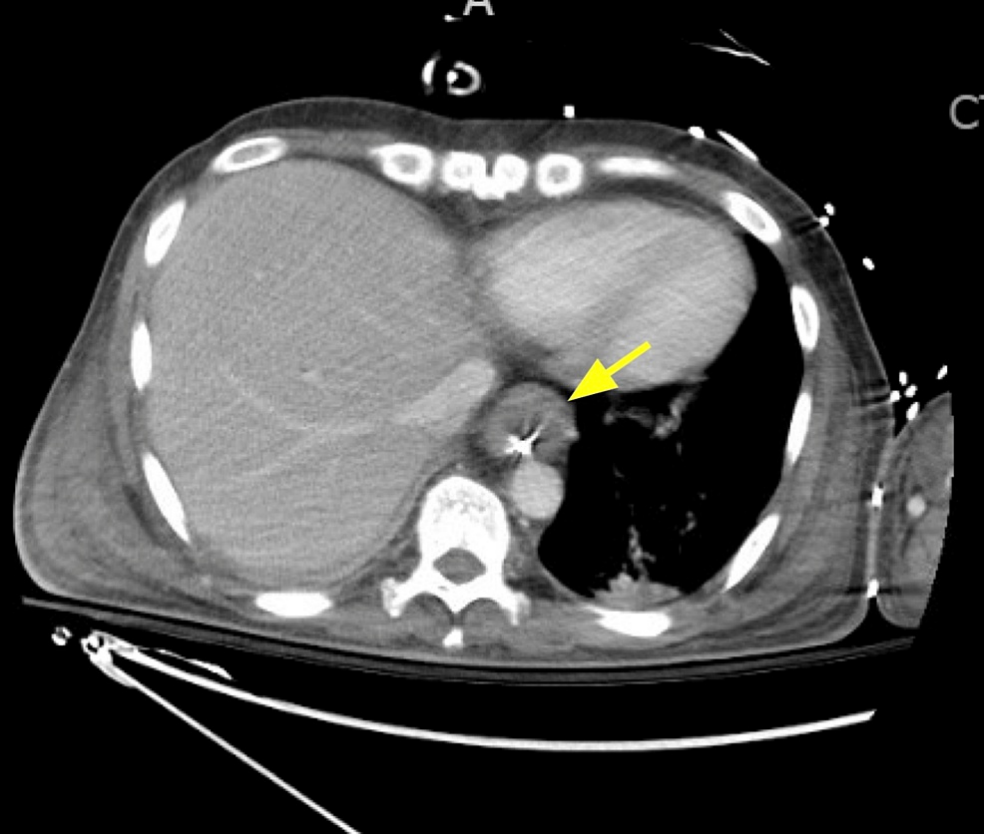
Computed Tomography of the Abdomen and Pelvis Demonstrated Lower Circumferential Esophageal Wall Thickening and Retroperitoneal Lymphadenopathy Arrow pointing to esophageal wall thickening

A 57-year-old male, never-smoker, with no known medical history, presented to our academic medical center from an outside hospital for continued evaluation and treatment of severe, new-onset, right-sided heart failure, which had not improved despite implantation of a ventricular assist device. His initial symptoms were that of progressively worsening dyspnea and cough of several weeks duration, with no other stated complaints. On physical examination, he was ill-appearing, tachycardic, and hypotensive. His lungs were clear to auscultation bilaterally. Uncontrolled bleeding was noted at peripheral intravenous access sites, and distal extremities were cold and dusky. The remainder of the clinical examination was unremarkable. Initial labs were notable for hemoglobin of 8.9 g/dL and a platelet count of 100,000 K/uL, as well as an elevated blood urea nitrogen, creatinine, and brain natriuretic peptide. Computed tomography pulmonary angiography was negative for pulmonary embolism. The patient presented on intravenous infusions of norepinephrine for refractory hypotension and heparin for ventricular assist device anticoagulation. He was started on vancomycin and piperacillin/tazobactam for concern of sepsis and continuous furosemide for diuresis. Throughout the course of his hospitalization, his hemoglobin and platelet count continued to decline to lows of 5.5 g/dL and 20,000 K/uL, respectively, and he ultimately required several transfusions of blood products. Further anemia workup revealed a low haptoglobin and elevated lactate dehydrogenase, total bilirubin, fibrinogen, and prothrombin time. Peripheral blood smear analysis revealed the presence of schistocytes, basophilic stippling, and Dohle bodies. Iron profile was notable for normal serum iron and total iron binding capacity and a markedly elevated ferritin of greater than 10,000 ng/mL. Vitamins B12 and folic acid levels were normal. The direct antiglobulin test was micropositive for C3 and negative for IgG, while the indirect Coombs test was negative, and ADAMTS13 activity was within normal limits. A bone marrow biopsy demonstrated that the marrow had been replaced by a malignant epithelial proliferation with intracellular mucin. The intramarrow tumor cell immunohistochemistry stains were positive for cytokeratin, CK7, CDX2, and CK20; faintly positive for GATA3; and negative for PSA, PSAP, and TTF1. Computed tomography of the abdomen and pelvis was performed to evaluate for occult intra-abdominal infection, which demonstrated lower circumferential esophageal wall thickening and retroperitoneal lymphadenopathy (Figure 1).

**Figure 2:**
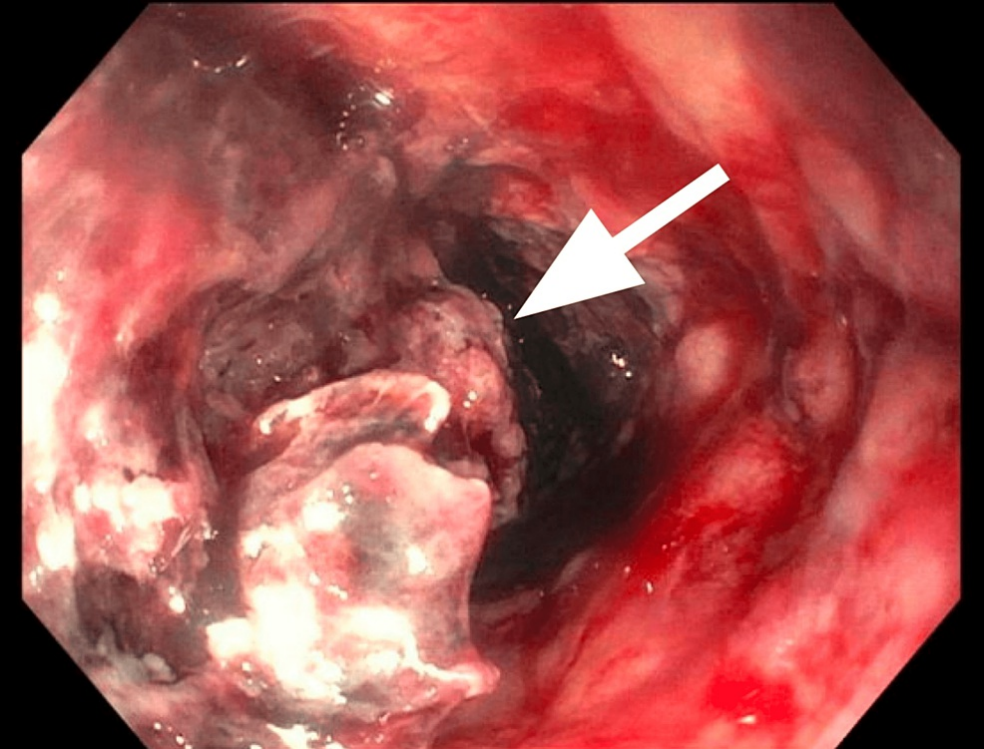
Upper Endoscopy with Esophageal Mass Arrow pointing to esophageal mucosa that was biopsied due to the friable, granular, and nodular appearance

He underwent upper endoscopy with an esophageal biopsy of esophageal mucosa that was notably friable, granular, and nodular (Figure 2).

Pathology results from the esophageal biopsy were consistent with adenocarcinoma. The patient continued to clinically deteriorate despite all interventions. Due to his rapidly progressive right heart failure and newly diagnosed metastatic GI malignancy, the diagnosis of PTTM was made. Given the universally fatal prognosis of PTTM the patient, family, and care team decided to pursue comfort care measures. The patient passed away shortly thereafter.

## Discussion

PTTM is a rare, under-reported, and poorly studied condition characterized by microscopic tumor cells, which embolize the pulmonary vasculature, resulting in pulmonary hypertension, right heart failure, and thrombotic microangiopathy. The exact mechanism of PTTM is not known. However, it is proposed that the interaction of tumor emboli with endothelial cells triggers local and sometimes systemic activation of pro-coagulant and fibrinolytic pathways, resulting in the consumption of coagulation factors and platelets [3]. This proposal is consistent with our patient's lab findings of resistant thrombocytopenia and elevated prothrombin time. Another proposed mechanism is the upregulation of the vascular endothelial growth factor system via increased tissue factor expression, which further drives the proliferation of vascular endothelial cells. The resulting intimal thickening often results in the shearing of erythrocytes, producing schistocytes as seen on peripheral smears [4]. There have been a number of primary cancers associated with PTTM, the most common being gastric adenocarcinomas, followed by breast, lung, and urothelial malignancies in decreasing prevalence [3]. In the literature review, only one case of esophageal cancer PTTM has been reported [5], making the case presented above especially rare. PTTM is primarily diagnosed postmortem. In cases where it is diagnosed antemortem, the median prognosis is only 16.2 days [2]. There have been several pharmacologic therapies identified that can potentially alter the clinical course of the disease, including advanced pulmonary antihypertensives, such as epoprostenol, sildenafil, and ambrisentan; antineoplastic medications such as imatinib, irinotecan, and cisplatin; and diuretics and steroids [3]. The efficacy of these medications has not  been adequately studied. The difficulty remains, however, of identifying the disease earlier in its progression. Patients with PTTM present most often with dyspnea (94%) and cough (85%), as in our case presented above [3]. Other symptoms include abdominal pain, fatigue, and weight loss. The diagnosis of PTTM is made on the basis of histopathology or clinical assessment. At this time, there is no standardized approach to diagnosing PTTM. As demonstrated in the presented case, a bone marrow biopsy discovered malignant cells, and computed tomography of the abdomen/pelvis was critical in identifying the patient's primary malignancy. Other helpful diagnostic tools include computed tomography pulmonary angiography, which will generally be negative for pulmonary embolism [2], and echocardiogram, which will often demonstrate significant pulmonary hypertension and right heart strain [6]. One case series submitted to the European Journal of Radiology suggests that lung-perfused blood volume images obtained by dual-energy computed tomography may provide a benefit in diagnosing PTTM antemortem [7].

## Conclusions

PTTM is a poorly identified disease with a devastatingly poor prognosis. Few treatments with unknown benefits have been identified to help treat PTTM. Thus, while earlier diagnosis may not fundamentally alter the course of PTTM, it may assist patients and clinicians in making more informed decisions regarding patient care. PTTM should be included in the differential diagnosis of patients with unexplained coagulopathy, rapidly progressive and severe cardiopulmonary failure, and diagnosed or suspected malignancy. Moreover, increased awareness and identification of PTTM can lead to a deeper comprehension of the disease’s underlying mechanisms, paving the way for innovative treatment approaches and ultimately improving patient outcomes.
